# Depression and Risk of Sudden Cardiac Death and Arrhythmias: A Systematic Review and Meta-Analysis

**DOI:** 10.31083/RCM36520

**Published:** 2025-08-29

**Authors:** Yao You, Yongmin Shi, Qingwen Yu, Xiyun Rao, Xuhan Tong, Ting Tang, Siqi Hu, Shenghui Zhang, Xingwei Zhang, Hu Wang, Mingwei Wang, Jiake Tang

**Affiliations:** ^1^Department of Cardiology, Affiliated Hospital of Hangzhou Normal University, Zhejiang Key Laboratory of Medical Epigenetics, School of Basic Medical Sciences, Hangzhou Institute of Cardiovascular Diseases, Engineering Research Center of Mobile Health Management System&Ministry of Education, Hangzhou Normal University, 310015 Hangzhou, Zhejiang, China; ^2^Department of Cardiology, Hangzhou Lin’an Fourth People’s Hospital, 311321 Hangzhou, Zhejiang, China; ^3^Department of Cardiology, Jiande First People’s Hospital, 311608 Hangzhou, Zhejiang, China

**Keywords:** depression, sudden cardiac death, arrhythmias, systematic review, meta-analysis

## Abstract

**Background:**

Depression is a highly prevalent mental disorder worldwide and is often accompanied by various somatic symptoms. Clinical studies have suggested a close association between depression and cardiac electrophysiological instability, particularly sudden cardiac death (SCD) and arrhythmias. Therefore, this review systematically evaluated the association between depression and the risks of SCD, atrial fibrillation (AF), and ventricular arrhythmias.

**Methods:**

This analysis was conducted in accordance with the Preferred Reporting Items for Systematic reviews and Meta-Analyses guidelines. The PubMed, Embase, Web of Science, China National Knowledge Infrastructure, VIP, and Wanfang databases were comprehensively searched to identify studies that indicated a correlation between depression and the risk of SCD and arrhythmias from database inception until April 10, 2025. Numerous well-qualified cohort studies were incorporated in this analysis. Correlation coefficients were computed using a random effects model. Statistical analyses were performed using Review Manager 5.4 and STATA 16.0.

**Results:**

A total of 20 studies were included in this meta-analysis. We explored the relationship between depression and SCD as well as arrhythmias. Of these diseases, SCD exhibited a statistically significant association with depression (hazard ratio (HR), 2.52, 95% confidence interval (CI): 1.82–3.49). Ventricular tachycardia (VT)/ventricular fibrillation (VF) was also significantly correlated with depression (HR): 1.38, 95% CI: 1.03–1.86). Depression was also considerably more likely to develop following AF. The results also indicated that AF recurrence (HR: 1.89, 95% CI: 1.54–2.33) was more significant than new-onset AF (HR: 1.10, 95% CI: 0.98–1.25).

**Conclusions:**

This study highlights a significant association between depression and elevated risks of SCD and arrhythmias, including both AF and VT/VF. These findings underscore the importance of incorporating mental health evaluation into comprehensive cardiovascular risk management strategies.

**The PROSPERO registration:**

CRD42024498196, https://www.crd.york.ac.uk/PROSPERO/view/CRD42024498196.

## 1. Introduction

Depression is an exceedingly prevalent mental disease globally. From 1990 to 2017, the global incidence of depression has markedly increased by 49.86% [1], thereby rendering it a major public health concern. Nearly one-third (34%) of adolescents are considered to possess a risk of clinical depression [2], while approximately one-eighth (13.3%) of elderly people, especially older women, have developed severe depression [3]. A recent study estimated an average prevalence of depression in inpatients of 12% [4], indicating that depression is always accompanied by somatic symptoms.

In addition to factors such as age, gender, and unhealthy lifestyles, several diseases are associated with depression. Recent international research has indicated that individuals exhibiting depressive symptoms face a markedly elevated risk of experiencing acute stroke, encompassing both ischemic and hemorrhagic subtypes [5]. Furthermore, pooled evidence from meta-analyses supports a robust association between depression and increased stroke incidence [6]. Similarly, depressive manifestations have been linked to a heightened likelihood of developing peripheral artery disease (PAD) [7]. It has also been suggested that depressed patients have impaired cardiac autonomic function and may be more susceptible to arrhythmias such as atrial or ventricular premature beats [8]. These risk factors can also contribute to cardiovascular disease development [9]. Several studies have explored the connection between cardiovascular diseases and depression.

These studies suggest that depression is associated with pan-vascular sclerosis and cardiac electrophysiologic disturbances. These findings imply that depression may be accompanied by underlying biological abnormalities, including chronic inflammation, lipid accumulation, and neurological dysfunction. When compared with the general population, patients with depression are more likely to develop atherosclerosis and experience major cardiac events [10]. In this study, we evaluated the correlation between depression and cardiovascular diseases, while the correlations of depression with atrial fibrillation (AF), ventricular tachycardia (VT), ventricular fibrillation (VF), and sudden cardiac death (SCD) were analyzed separately.

## 2. Materials and Methods

### 2.1 Study Design

This systematic review was conducted according to the Preferred Reporting Items for Systematic Reviews and Meta-Analysis guidelines (CRD42024498196) [11]. This study required no ethics committee approval as it was based on secondary research conducted using the existing literature.

### 2.2 Eligibility Criteria

The meta-analysis population included depression patients, who were diagnosed using related mental scales, such as the self-rating Depression Scale (DEPS), Health Questionnaire (PHQ)-9, and Hospital Anxiety and Depression Scale-D, in line with international standards. Cohort studies were considered eligible. Cross-sectional studies, descriptive research, animal studies, and *ex-vivo* studies were excluded from the analysis.

### 2.3 Literature Search

Relevant trials were identified by searching PubMed, Web of Science, Embase, and China National Knowledge Infrastructure databases, VIP, and Wanfang databases up to April 10, 2025, and then screening the references of retrieved studies. These studies were retrieved based on keywords and medical subject headings. The main search strategy applied was as follows: (“depressive symptoms” or “depression” or “depressive disorder”) and (“sudden cardiac death” or “arrhythmias” or “ventricular tachycardia” or “ventricular fibrillation” or “VT/VF” or “atrial fibrillation”). Detailed search strategy is depicted in **Supplementary Table 1**.

### 2.4 Study Selection

Literature screening was conducted in three stages: (1) studies related to the topic were selected after screening the article titles and abstracts. (2) Several full texts were browsed to identify literature that might match the topic. Studies meeting all of the following criteria were included: Cohort studies published in full text; literature assessing the correlation between depression and risk of SCD or arrhythmia; depression and SCD or arrhythmia risk defined according to clinical criteria; articles reporting the effect size, which is the primary outcome indicator of SCD or arrhythmia. (3) Studies without any results of interest or those meeting any of the exclusion criteria were excluded. Study selection was independently conducted by two researchers, and any potential disputes were resolved.

Depression was defined as elevated depressive symptoms of depression measured by a validated questionnaire, structured interview, or history of depression, [International Classification of Diseases (ICD)-10: F32.0–32.9, F33.0–33.3, F33.8, F33.9, F34.1, and F41.2]. SCD was defined as death, including cardiac arrest, occurring within 1 h of the symptom onset (ICD-10: I05–I25, I30–I52). Arrhythmias included only VT/VF or AF, which were defined according to the exact clinical criteria. AF was categorized as new-onset AF and recurrent AF, whereas VT/VF analysis did not include premature ventricular contractions.

### 2.5 Data Extraction

The following additional information was extracted from all included studies by using a pre-designed extraction form. These data were incorporated into a Microsoft Excel spreadsheet. The information included the name of the author, year, name of the study area, study design, characteristics of the participants, the number of participants, patient gender, patient age, diagnostic criteria, duration of follow-up, reported outcomes, and confounders adjusted. The two researchers collected the data and resolved any potential differences arising after discussion with another author of the present study.

### 2.6 Quality Assessment

The Newcastle-Ottawa Scale was used to separately evaluate the cohort studies [11]. The scale ranges from 1 to 9 and assesses the quality of cohort studies based on the study selection, between-group comparability, and outcome assessment. Past studies that scored more than 6 were categorized as high-quality studies. Two review authors (Yao You and Siqi Hu) independently completed the literature assessment. These authors were blinded to each other’s scores.

### 2.7 Sensitivity and Publication Bias

The funnel plot was employed to assess the existence of publication bias in the meta-analysis. The Egger test was conducted to estimate its asymmetry. The sensitivity analysis was performed by excluding one study at a time.

### 2.8 Statistical Analysis

Data analysis was performed using Stata 16 (StataCorp LP, College Station, TX, USA) and Review Manager 5.4 (RevMan Development Core Team, Oxford, England), with a two-sided *p* value of 0.05 defined as being statistically significant. The odds ratio (OR) or hazard ratio, along with their associated 95% confidence intervals (95% CI), were used as the relevant coefficients for evaluating relevance. For studies that categorized the depression index into quartiles, the risk ratio for disease occurrence was extracted for subjects with the highest levels of depression when compared to those with the lowest levels of depression. The heterogeneity of the included cohort studies was assessed using the I^2^ statistic [12]. The inconsistency index (I^2^) was calculated to determine publication heterogeneity and values of >50% suggested significant heterogeneity, values of >50% indicated significant heterogeneity, using a random effects model, and I^2^ <50% used a fixed-effects model. Predefined subgroup and meta-regression analyses were conducted to evaluate the effect of study characteristics (including the percentage of males, sample size, population, and exposure measurement) on the association between depression and new-onset AF.

### 2.9 Certainty of Evidence

The Grading of Recommendations Assessment, Development, and Evaluation principles were followed to assess the certainty of the evidence. Considering the limitations, inconsistency, imprecision, and publication bias, the facts were classified into four levels, namely very low, low, moderate, and high.

## 3. Results

### 3.1 Study Selection

**Fig. 1.**
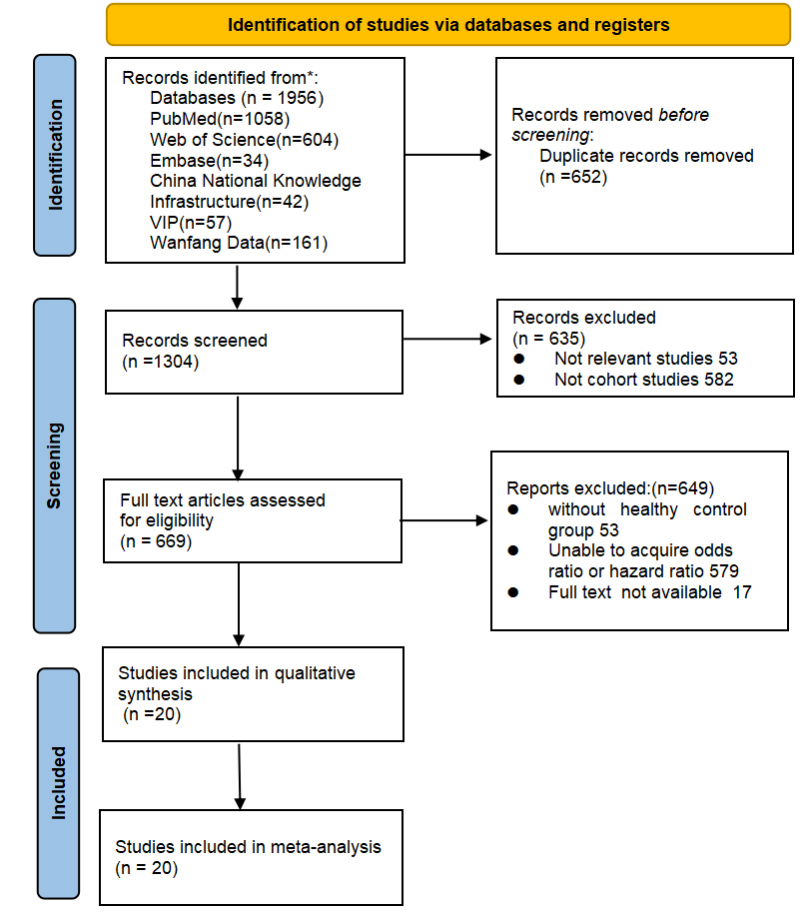
**Flow diagram depicting literature retrieval**. *Databases searched include PubMed, Web of Science, Embase, CNKI, VIP, and Wanfang.

The document screening process is depicted in Fig. 1. In total, 1956 articles were retrieved from the databases. A total of 1304 articles were selected after removing the duplicate articles. Of these, 669 studies were searched with reference to the full text. Subsequently, 649 articles were eliminated because of the absence of available correlation coefficients. Thus, this meta-analysis comprised 20 studies, which included 4 SCD-related studies and 16 arrhythmia-related studies.

### 3.2 Characteristics of Studies Included in the Meta-Analysis

The included studies are shown in Table 1 (Ref. [13, 14, 15, 16, 17, 18, 19, 20, 21, 22, 23, 24, 25, 26, 27, 28, 29, 30, 31, 32]), and information includes author name, year, study area name, study design, participant characteristics, number of participants, patient gender, patient age, diagnostic criteria, duration of follow-up, reported outcomes, and adjusted for confounders. Most of these studies are sourced from the United States and Europe, only three studies were from Asia and used psychological scales such as the Severity of Dependence Scale, Beck’s Depression Inventory, and DEPS. In total, 10,808,101 subjects were included in these 20 cohort studies. The mean age ranged from 46.99 to 78, and the proportion of male patients ranged from 0% to 83.2%.

**Table 1.** **Characteristics of Studies Included in the Meta-Analysis**.

Author and year	Region/Country	Study design	Characteristics of participants	Number of participants	Male (%)	Age (yr)	Measure	FU (yr)	Outcomes reported	Adjustment
Irvine *et al*. [13] 1999	Canada	Cohort study	Patients after MI	634	82.8	63.8 ± 10.8	BDI score ≥10	2	SCD 34	MI CHF
Luukinen *et al*. [14] 2003	Northern Finland	Cohort study	Population aged >70 years participating in the Depressive Symptoms Questionnaire	915	36.7	78 ± 5	SZDRS ≥28 (19.1)	8	SCD 38	Male sex, history of MI, tablet- or insulin-treated diabetes mellitus, depressive symptoms
Whang *et al*. [15] 2009	USA	Cohort study	Women without prior coronary heart disease, stroke, or cancer	75,718	0	58.4	MHI <53 (6.0) Antidepressant (6.7)	8	SCD 99	Age, beginning year of follow-up, smoking, MI, alcohol intake, menopausal and postmenopausal hormone, aspirin use, multivitamin use, vitamin E tabl use, hypercholesterolemia, family history of MI, history of stroke, n-3 fatty acid intake, alpha-linolenic acid intake, moderate/vigorous physical activity, nonfatal CHD, hypertension, diabetes
Lahtinen *et al*. [16] 2018	USA	Cohort study	Patients with angiographically documented CAD	1928	DEPS Quartile 1st: 80% 4th: 61%	66	DEPS ≥8	6.3	SCD 49	Age, sex, body mass index, type 2 diabetes, Canadian Cardiovascular Society grading of angina pectoris, left ventricular ejection fraction, the use of psychotropic medication, and leisure-time physical activity.
Whang *et al*. [17] 2005	USA	Cohort study	ICD patients for whom baseline CES-D scale scores were available	645	81.7	64.1	CES-D ≥16 (17.9)	1	VT/VF 103	Age, sex, number of prior ICD discharges, time from ICD implant to study enrollment, cardiac arrest, CAD, angina class, CHF class, LVEF, smoking, alcohol use, selective serotonin reuptake inhibitor use, use of ACEI or ARB
Watkins *et al*. [18] 2006	USA	Cohort study	Patients with CAD	940	69.6	62	BDI ≥10 (28.0)	3	VT/VF 97	LVEF, age, sex, minority status, history of arrhythmias of arrhythmias
Huffman *et al*. [19] 2008	USA	Cohort study	Patients with a preliminary diagnosis of MI	129	79.8	62.2	DIS 13.2	NA	VT/VF 51	Prior MI, peak troponin T, LVEF
Frasure-Smith *et al*. [20] 2009	NA	Cohort study	Patients with AF and CHF	974	82.3	66	BDI-II ≥14 (32.0)	1.6	VT/VF 111	Age, marital status, cause of CHF, creatinine level, LVEF, paroxysmal AF, previous AF hospitalization, previous electrical conversion, baseline medications
Van den Broek *et al*. [21] 2009	Netherlands	Cohort study	Patients who underwent ICD	391	80.6	62.3	BDI ≥10 (35.3)	1	VT/VF 75	Sex, race, antidepressant use, diabetes mellitus, MI, LVEF, QRS duration, PR and QT intervals, history of VT/VF
Suzuki *et al*. [22] 2011	Japan	Cohort study	Patients hospitalized with CVD	505	72	61	SDS ≥60 (21.6)	1.1	VT 16	NA
Turagam *et al*. [23] 2012	USA	Cohort study	Implanted ICD patients with a history of depression	361	64.6	76.2	History of depression 23.0	2.5	VT/VF 236	NA
Lange and Herrmann-Lingen [24] 2007	Germany	Cohort study	Patients with atrial fibrillation and flutter	54	68.5	66.1	HADS >7 (24.1)	0.2	AF 27	LVEF, age, left atrial diameter, negative affectivity, AF duration
Tully *et al*. [25] 2011	Australia	Cohort study	Patients undergoing first-time CABG surgery	226	83.2	63.1	DASS ≥10 NA	NA	AF 56	Age, urgent procedure, LVEF, mitral incompetence, preoperative stress, preoperative anxiety
Yu *et al*. [26] 2012	China	Cohort study	Patients with a diagnosis of persistent AF	164	67.4	58.3	SDS ≥50 NA	1	AF 17	NA
Whang *et al*. [27] 2012	USA	Cohort study	Women without a history of cardiovascular disease or AF	30,746	0	59	MHI <53 (6.8) Antidepressant (7.1)	10.4	AF 771	Age, race, BMI, hypertension, diabetes, hypercholesterolemia, smoking, alcohol intake, kilocalories from exercise, treatment
Efremidis *et al*. [28] 2014	Greece	Cohort study	Patients with paroxysmal AF	57	59.6	56.9	BDI ≥14 NA	0.7	AF 16	Age, sex, BMI, diabetes, hypertension
Feng *et al*. [29] 2020	Norway	Cohort study	Participants with no history of AF at baseline	37,402	43.5	53.4 ± 15.2	HADS-D ≥11	8.1	AF 1433	Age, sex, weight, height smoking status, occupation, marital status, physical activity, alcohol consumption, chronic disorders, and metabolic components
Kim *et al*. [30] 2022	South Korean	Cohort study	Participants with no history of AF at baseline	5,031,222	55.1	46.99 (14.06)	ICD-10	10	New-onset AF 78,262	Age, sex, BMI, smoking status, alcohol consumption status, physical activity, income level, diabetes mellitus, hypertension, dyslipidemia, heart failure, and thyroid disease
Fu *et al*. [31] 2024	USA	Cohort study	Patients >50 years of age with a history of hospitalization for heart failure	784	50	NA	PHQ-9 ≥10	3	AF 63	Age, sex, NYHA class; BMI; current smoking; previous hospitalization for CHF; previous myocardial infarction; previous stroke; hypertension; peripheral vascular disease; diabetes mellitus; ACEI/ARB; diuretic, anti-depression; eGFR; COPD, previous PCI, previous CABG, randomization
Smith *et al*. [32] 2025	Swedish	Cohort study	Patients aged >40 years	5,624,306	48.8	53	Clinical diagnosis	3.3	AF 453,280	Sex, highest attained level of education, county of residence, and entrance year in the study

MI, acute myocardial infarction; BDI, Beck Depression Inventory; CHF, congestive heart failure; SZDRS, Short Zung Depression Rating Scale; N/A, not available; MHI, Mental Health Index; BMI, body mass index; CHD, coronary heart disease; CAD, coronary artery disease; ICD, implanted cardioverter defibrillator; CES-D, Center For Epidemiologic Studies Depression Scale; VF, ventricular fibrillation; LVEF, left ventricular ejection fraction; ACEI, angiotensin-converting enzyme inhibitors; ARB, angiotensin receptor blocker; CVD, cardiovascular disease; SDS, self-rating depression scale; HADS, hospital anxiety and depression scale; CABG: coronary artery bypass grafting; DASS, depression anxiety stress scales; FU, follow-up; MHI, Mental Health Inventory; BDI, Beck Depression Inventory; PHQ, Patient Health Questionnaire; NYHA, New York Heart Association; eGFR, Estimated Glomerular Filtration Rate; COPD, Chronic obstructive pulmonary disease; PCI, Percutaneous coronary intervention; CABG, Coronary Angioplasty Bypass Grafting.

### 3.3 Quality Evaluation

The 20 studies [13, 14, 15, 16, 17, 18, 19, 20, 21, 22, 23, 24, 25, 26, 27, 28, 29, 30, 31, 32] included in this meta-analysis were cohort studies. Quality was assessed using the Newcastle-Ottawa Scale (maximum score of 9). The results revealed that four studies scored 9, four studies scored 8, eight studies scored 7, and four studies scored 6. Therefore, all included cohort studies were recognized as high quality (Table 2). Most of these studies had a long follow-up period (≥1 year), but 2 studies [24, 28] had a follow-up period of <1 year. A total of 17 studies [13, 14, 15, 16, 17, 18, 19, 20, 21, 24, 25, 27, 28, 29, 30, 31, 32] were adjusted for potential confounders through exclusion criteria or statistical adjustments; 3 studies [22, 23, 26] did not adjust or did not report their adjusted variables.

**Table 2.** **Details of quality evaluation via the Newcastle–Ottawa Scale**.

Study	Selection	Comparability	Outcome/Exposure	NOS score
Irvine *et al*.	3	1	2	6
Luukinen *et al*.	4	1	3	8
Whang *et al*.	4	2	3	9
Minna Lahtinen MSc	3	2	3	8
Whang *et al*.	3	2	2	7
Watkins *et al*.	3	2	3	8
Huffman *et al*.	3	1	2	6
Frasure-Smith *et al*.	3	2	2	7
Van den Broek *et al*.	3	2	2	7
Suzuki *et al*.	3	1	3	7
Turagam *et al*.	3	1	3	7
Lange *et al*.	3	2	2	7
Tully *et al*.	3	2	1	6
Yu *et al*.	3	1	2	6
Whang *et al*.	4	2	3	9
Efremidis *et al*.	3	2	2	7
Tingting Feng	4	2	3	9
Yun Gi Kim	4	2	3	9
Fu Y	3	2	3	7
Smith C	4	1	3	8

The NOS consists of eight items categorized into three aspects. Each numbered item can score one star if the study is eligible. A maximum of four stars can be awarded for selection, two stars for comparability, and three stars for outcome or exposure. NOS, Newcastle-Ottawa scale. Note: The “Study” column corresponds to the “Author and year” information presented in Table 1.

### 3.4 Depression and SCD

**Fig. 2.**
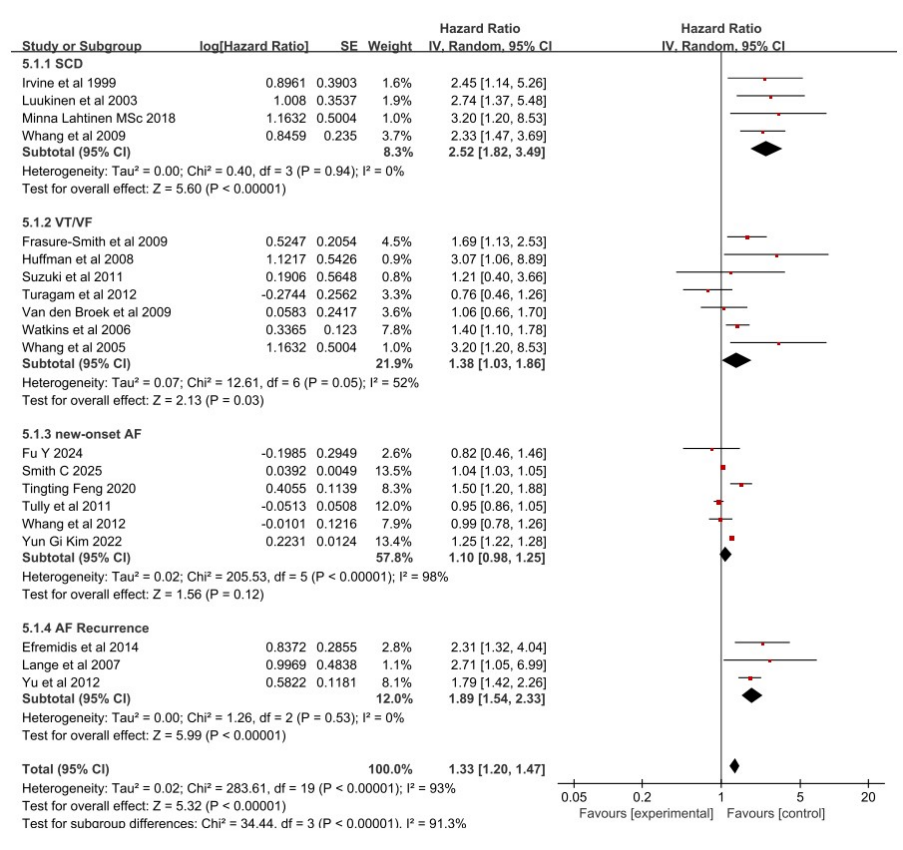
**Forest plots depicting depression and risk of sudden cardiac death and arrhythmias**. SCD, sudden cardiac death; VT/VF, ventricular tachycardia/ventricular fibrillation; AF, atrial fibrillation; CI, confidence interval; SE, standard error; IV, inverse variance; df, degrees of freedom.

Four cohort studies [13, 14, 15, 16] involving a total of 79,195 participants were included for SCD analysis. The results of the meta-analysis are shown in Fig. 2. Depression was associated with an increased risk of SCD (HR: 2.52, 95% CI: 1.82–3.49, I^2^ = 0%, *p* < 0.01).

### 3.5 Depression and VA/VF

**Fig. 3.**
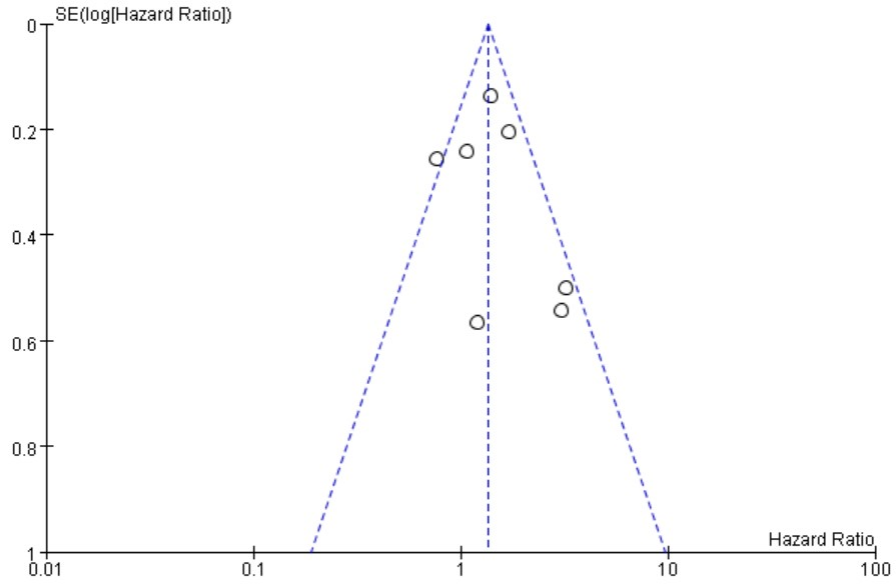
**Funnel plot for the publication bias underlying the meta-analysis of the association between depression and VT/VF**.

Seven cohort studies [17, 18, 19, 20, 21, 22, 23] involving a total of 3945 subjects were included. Depression exhibited a significant association with an increased risk of VT/VF (HR: 1.38, 95% CI: 1.03–1.86), I^2^ = 52%, *p* = 0.03; Fig. 2). For results exhibiting moderate heterogeneity, sensitivity analyses were performed, with each study excluded showing consistent results with no significant change in heterogeneity or combined HR values (HR: 1.28–1.47, *p* < 0.01). This finding proves the stability of the findings (**Supplementary Fig. 1**). Visual inspection of the funnel plot exhibited symmetry, indicating a low risk of publication bias (Fig. 3), while Egger’s test (*p* = 0.993), indicated no risk of bias (**Supplementary Fig. 2**).

### 3.6 Depression and AF

Fig. 2 displays the association between AF and depression. Six cohort studies [25, 27, 29, 30, 31, 32] involving 10,724,686 subjects were analyzed to demonstrate the relationship between new-onset AF and depression. In addition, the remaining 3 outcome indicators [24, 26, 28] were recurrent AF, involving a total of 275 subjects. The results show that individuals who had recurrent AF exhibited an increased risk of depression (HR: 1.89, 95% CI: 1.54–2.33, I^2^ = 0%, *p* < 0.01) when compared with those who had new AF (HR: 1.10, 95% CI: 0.98–1.25, I^2^ = 98%, *p* = 0.12). Among them, the results for new-onset AF were not statistically significant and displayed a high degree of heterogeneity. Meta-regression and subgroup analyses of the new-onset AF group by sample size, proportion of males, population, and exposure measurement, did not show any significant effect (*p* > 0.05, **Supplementary Table 2**). Significant heterogeneity was found in these subgroups, suggesting that these factors were not a major source of heterogeneity.

Using the one-by-one exclusion method, we determined a significant change in heterogeneity after deleting Yun Gi Kim, from 98% to 72%, HR: 1.06 (95% CI: 0.94–1.19), albeit the results were not statistically significant (*p* = 0.36). The sensitivity analysis chart is shown in **Supplementary Fig. 3**.

## 4. Discussion

This meta-analysis, based on 20 cohort studies and over 10 million participants, provides compelling evidence for a significant association between depression and the risk of SCD, VT/VF, and AF. Notably, the association between depression and new-onset AF was not statistically significant. The most robust association was observed between depression and SCD (HR: 2.52), suggesting that individuals with depressive symptoms are more than twice as likely to experience SCD compared to non-depressed individuals. Depression was also moderately associated with VT/VF (HR: 1.38) and recurrent AF (HR: 1.89), highlighting its broader implications for electrophysiological instability.

The association between depression and adverse cardiac electrophysiological outcomes is likely multifactorial and bidirectional, involving behavioral, autonomic, neurohormonal, and inflammatory pathways. Depression has been shown to disrupt autonomic balance, characterized by increased sympathetic tone and reduced parasympathetic activity [33]. Heart rate variability (HRV), a surrogate marker of autonomic regulation, is significantly reduced in depressed patients, a finding correlated with heightened susceptibility to ventricular arrhythmias and SCD [34]. The loss of vagal protection may also facilitate atrial electrical instability, thereby promoting recurrent AF. Depression is a heterogeneous disorder with different subtypes having diverse effects on the autonomic nervous system function and cardiac electrophysiology. The internalizing depressive subtype (characterized by low mood, withdrawal, and a lack of pleasure) is more often associated with reduced HRV, suggesting diminished parasympathetic (vagal) nerve activity [35]. The agitated or comorbid anxiety subtype of depression is usually associated with increased sympathetic nerve activity and prolonged QT interval, thereby increasing the risk of ventricular arrhythmias [36].

In addition to autonomic imbalance, chronic low-grade systemic inflammation plays a central role. Depressive states are characterized by elevated levels of inflammatory cytokines, such as interleukin-6, tumor necrosis factor-alpha, and C-reactive protein, which have been demonstrated to induce endothelial dysfunction, promote myocardial fibrosis, and enhance atrial and ventricular arrhythmogenicity through structural and electrical remodeling [37, 38]. Furthermore, hypothalamic–pituitary–adrenal axis dysregulation in depression causes hypercortisolemia and heightened catecholamine release, further aggravating autonomic and inflammatory disturbances [39, 40]. Patients with depression are more likely to engage in unhealthy behaviors such as smoking, physical inactivity, poor diet, and medication non-adherence, all of which can exacerbate cardiovascular risks. Moreover, sleep disturbances—common in depressed individuals—are known triggers for AF onset and SCD, especially during nocturnal sympathetic surges [41]. Certain antidepressants, particularly tricyclics, and some SSRIs, may prolong the QT interval and increase the risk of torsades de pointes or ventricular arrhythmias [42]. Although it has not been directly addressed in most included studies, this pharmacological factor warrants consideration in clinical interpretation.

The differential effect observed between recurrent and new-onset AF is notable. Depression was significantly associated with AF recurrence but not with incident AF. One possible explanation is that recurrent AF patients may already have structural atrial remodeling and autonomic vulnerability, both of which can be exacerbated by depression. Moreover, recurrent episodes may intensify psychological stress, creating a vicious cycle. The high heterogeneity (I^2^ = 98%) in new-onset AF studies suggests substantial methodological and population differences, including inconsistent definitions of depression, variations in follow-up duration, and differences in underlying cardiovascular risk profiles. Sensitivity analysis showed that the exclusion of a single study [30] reduced heterogeneity, but statistical significance was still not achieved.

Our findings underscore the need to integrate mental health screening, particularly for depression, into cardiovascular risk stratification models. For patients with established cardiovascular disease, depression should not only be considered a comorbidity but a potential prognostic marker for fatal arrhythmias and SCD. Psychotherapeutic and pharmacological treatment of depression may have cardioprotective effects. Previous studies demonstrated that effective depression management—especially cognitive behavioral therapy (CBT)—can reduce arrhythmic events and cardiovascular mortality, particularly among younger patients (<60 years) [43]. In clinical practice, CBT remains the first-line psychotherapeutic intervention, with well-documented benefits in both mood regulation and modulation of autonomic function [44]. Additional options such as interpersonal therapy (IPT) and mindfulness-based cognitive therapy (MBCT) may be beneficial, especially in patients with recurrent or treatment-resistant depression [45]. From a pharmacological perspective, selective serotonin reuptake inhibitors (SSRIs)—particularly sertraline and escitalopram—are preferred in patients with cardiovascular disease owing to their favorable safety profiles and low arrhythmogenic potential [46, 47]. Conversely, tricyclic antidepressants (TCAs) and monoamine oxidase inhibitors (MAOIs) should generally be avoided in this population owing to their known proarrhythmic effects [48]. However, large-scale interventional studies are needed to verify these benefits.

Despite the strengths of this meta-analysis, several limitations should be acknowledged. First, the number of studies available for certain key outcomes—specifically SCD and recurrent AF—was relatively small, which may compromise the statistical robustness and precision of the pooled estimates. Second, the majority of included cohorts were drawn from high-income Western countries, thereby limiting the generalizability of the findings to Asian populations and other low- and middle-income regions where epidemiological and healthcare contexts may differ. Third, the diagnosis of depression varied across studies, with different psychometric tools such as the PHQ-9, HADS, and BDI applied inconsistently, potentially introducing classification bias. Additionally, all included studies were observational in design, which restricts the ability to infer causality and leaves open the possibility of residual confounding, even when adjustments were reported. Lastly, although no major publication bias was detected through Egger’s test, the small number of studies contributing to some endpoints may have reduced the sensitivity of this assessment.

## 5. Conclusions

Our study findings demonstrated that depression was significantly correlated with SCD and cardiovascular diseases, including VT/VF and AF. Psychotherapeutic interventions may be a crucial player in the health management of patients with cardiovascular diseases.

## Availability of Data and Materials

Not applicable.

## Supplementary Material

Supplementary material associated with this article can be found, in the online version, at https://doi.org/10.31083/RCM36520.
