# Supplementary material for: Depression and Risk of Sudden Cardiac Death and Arrhythmias: A Systematic Review and Meta-Analysis
Source: Rev Cardiovasc Med. 2025 Aug 29;26(8):36520. doi: 10.31083/RCM36520 (PMC12415728; doi:10.31083/RCM36520)
Supplement: Supplementary file 1 [file 2153-8174-26-8-36520-s1.zip › SupplementaryMaterial.docx]

**Supplementary Table 1:** Detailed description of the search strategy.

| **PubMed** | |
| --- | --- |
| #1 | depression[MeSH Terms] OR Depressive Symptom[Title/Abstract] OR  Depressive Symptoms[Title/Abstract] |
| #2 | Atrial fibrillation[Title/Abstract] OR Atrial Fibrillations[Title/Abstract] OR Fibrillation, Atrial OR Fibrillations, Atrial[Title/Abstract] OR Auricular Fibrillation[Title/Abstract] OR Auricular Fibrillations[Title/Abstract] OR Persistent Atrial Fibrillation[Title/Abstract] OR Atrial Fibrillation, Persistent[Title/Abstract] OR Atrial Fibrillations, Persistent[Title/Abstract] OR Fibrillation, Persistent Atrial[Title/Abstract] OR Fibrillations, Persistent Atrial[Title/Abstract] OR Persistent Atrial Fibrillations[Title/Abstract] OR Paroxysmal Atrial Fibrillation[Title/Abstract] OR Atrial Fibrillation, Paroxysmal[Title/Abstract] OR Atrial Fibrillations, Paroxysmal [Title/Abstract] OR Fibrillation, Paroxysmal Atrial [Title/Abstract] OR Fibrillation, Paroxysmal Atrial [Title/Abstract] OR Paroxysmal Atrial Fibrillations[Title/Abstract] |
| #3 | Ventricular tachycardia[Title/Abstract] OR Ventricular Tachycardias[Title/Abstract] OR Ventricular Tachycardia[Title/Abstract] OR Idiopathic Ventricular Tachycardia[Title/Abstract] OR Idiopathic Ventricular Tachycardias[Title/Abstract] OR Tachycardia, Idiopathic Ventricular [Title/Abstract] OR Ventricular Tachycardia, Idiopathic[Title/Abstract] OR Ventricular Tachycardia, Idiopathic[Title/Abstract] OR Nonsustained Ventricular Tachycardias[Title/Abstract] OR Nonsustained Ventricular Tachycardias[Title/Abstract] OR Nonsustained Ventricular Tachycardias[Title/Abstract] OR Ventricular Tachyarrhythmias[Title/Abstract] OR Tachyarrhythmia, Ventricular[Title/Abstract] OR Tachyarrhythmia, Ventricular[Title/Abstract] OR Fibrillations, Ventricular[Title/Abstract] OR Fibrillation, Ventricular[Title/Abstract] OR Ventricular Fibrillations[Title/Abstract] |
| #4 | Sudden cardiac death[Title/Abstract] OR Cardiac Sudden Death[Title/Abstract] OR Death, Sudden Cardiac[Title/Abstract] |
| #5 | #2 OR #3 OR #4 |
| #6 | #1 AND #5 |
| **Embase** | |
| #1 | Depression/exp OR  Depressive Symptom:ab,kw,ti OR  Depressive Symptoms |
| #2 | Atrial fibrillation:ab,kw,ti OR Atrial Fibrillations:ab,kw,ti OR Fibrillation, Atrial:ab,kw,ti OR Fibrillations, Atrial:ab,kw,ti OR Auricular Fibrillation:ab,kw,ti OR Auricular Fibrillations:ab,kw,ti OR Persistent Atrial Fibrillation:ab,kw,ti OR Atrial Fibrillation, Persistent:ab,kw,ti OR Atrial Fibrillations, Persistent:ab,kw,ti OR Fibrillation, Persistent Atrial:ab,kw,ti OR Fibrillations, Persistent Atrial:ab,kw,ti OR Persistent Atrial Fibrillations:ab,kw,ti OR Paroxysmal Atrial Fibrillation:ab,kw,ti OR Atrial Fibrillation, Paroxysmal:ab,kw,ti OR Atrial Fibrillations, Paroxysmal:ab,kw,ti OR Fibrillation, Paroxysmal Atrial :ab,kw,ti OR Fibrillation, Paroxysmal Atrial :ab,kw,ti OR Paroxysmal Atrial Fibrillations |
| #3 | Ventricular tachycardia:ab,kw,ti OR Ventricular Tachycardias:ab,kw,ti OR Ventricular Tachycardia:ab,kw,ti OR Idiopathic Ventricular Tachycardia:ab,kw,ti OR Idiopathic Ventricular Tachycardias:ab,kw,ti OR Tachycardia, Idiopathic Ventricular :ab,kw,ti OR Ventricular Tachycardia, Idiopathic:ab,kw,ti OR Ventricular Tachycardia, Idiopathic:ab,kw,ti OR Nonsustained Ventricular Tachycardias:ab,kw,ti OR Nonsustained Ventricular Tachycardias:ab,kw,ti OR Nonsustained Ventricular Tachycardias:ab,kw,ti OR Ventricular Tachyarrhythmias:ab,kw,ti OR Tachyarrhythmia, Ventricular:ab,kw,ti OR Tachyarrhythmia, Ventricular:ab,kw,ti OR Fibrillations, Ventricular:ab,kw,ti OR Fibrillation, Ventricular:ab,kw,ti OR Ventricular Fibrillations |
| #4 | Sudden cardiac death:ab,kw,ti OR Cardiac Sudden Death:ab,kw,ti OR Death, Sudden Cardiac:ab,kw,ti |
| **Web of Science** | |
| #1 | TS=(Depression/exp OR  Depressive Symptom OR  Depressive Symptoms) |
| #2 | TS=(Atrial fibrillation OR Atrial Fibrillations OR Fibrillation, Atrial OR Fibrillations, Atrial OR Auricular Fibrillation OR Auricular Fibrillations OR Persistent Atrial Fibrillation OR Atrial Fibrillation, Persistent OR Atrial Fibrillations, Persistent OR Fibrillation, Persistent Atrial OR Fibrillations, Persistent Atrial OR Persistent Atrial Fibrillations OR Paroxysmal Atrial Fibrillation OR Atrial Fibrillation, Paroxysmal OR Atrial Fibrillations, Paroxysmal OR Fibrillation, Paroxysmal Atrial OR Fibrillation, Paroxysmal Atrial OR Paroxysmal Atrial Fibrillations) |
| #3 | TS=(Ventricular tachycardia OR Ventricular Tachycardias OR Ventricular Tachycardia OR Idiopathic Ventricular Tachycardia OR Idiopathic Ventricular Tachycardias OR Tachycardia, Idiopathic Ventricular OR Ventricular Tachycardia, Idiopathic OR Ventricular Tachycardia, Idiopathic OR Nonsustained Ventricular Tachycardias OR Nonsustained Ventricular Tachycardias OR Nonsustained Ventricular Tachycardias OR Ventricular Tachyarrhythmias OR Tachyarrhythmia, Ventricular OR Tachyarrhythmia, Ventricular OR Fibrillations, Ventricular OR Fibrillation, Ventricular OR Ventricular Fibrillations) |
| **China National Knowledge Infrastructure databases** | |
| #1 | SU= (depression OR depressive syndrome OR depression, neurosis OR neurotic depression OR melancholia OR unipolar depression OR depressive neurosis OR depression, endogenous OR endogenous depression OR endogenous depression) |
| #2 | SU= (AF OR AF transient OR AF persistent OR atrial fibrillation OR VT OR VT OR accelerated idioventricular rhythm OR torsades de pointes OR VT OR ventricular fibrillation OR ventricular fibrillation OR sudden cardiac death OR cardiac arrest OR sudden cardiac death) |
| #3 | #1 AND #2 |
| **VIP** | |
| #1 | M= (depression OR depressive syndrome OR depression, neurosis OR neurotic depression OR melancholia OR unipolar depression OR depressive neurosis OR depression, endogenous OR endogenous depression OR endogenous depression) |
| #2 | M= (AF OR AF transient OR AF persistent OR atrial fibrillation OR VT OR VT OR accelerated idioventricular rhythm OR torsades de pointes OR VT OR ventricular fibrillation OR ventricular fibrillation OR sudden cardiac death OR cardiac arrest OR sudden cardiac death) |
| #3 | #1 AND #2 |
| **Wanfang databases** | |
| #1 | depression OR depressive syndrome OR depression, neurosis OR neurotic depression OR melancholia OR unipolar depression OR depressive neurosis OR depression, endogenous OR endogenous depression OR endogenous depression |
| #2 | AF OR AF transient OR AF persistent OR atrial fibrillation OR VT OR VT OR accelerated idioventricular rhythm OR torsades de pointes OR VT OR ventricular fibrillation OR ventricular fibrillation OR sudden cardiac death OR cardiac arrest OR sudden cardiac death |
| #3 | #1 AND #2 |

**Supplementary Fig. 1**. Sensitivity analyses of association between depression and VT/VF by omitting one study at once

**Supplementary Fig. 2.** Egger’s test diagrams for depression and VT/VF

**Supplementary Table 2** Subgroup analysis of the meta-analysis for the risk of new-onset.

| Subgrouped by | Effect value HR (95% CI) | I-squared (%) | \| P overall effect \|  \| \| --- \| --- \| | P interaction |
| --- | --- | --- | --- | --- | --- | --- |
| Male%  ≥ 50%  < 50% | 1.10 ( 0.98,1.25 )  1.05 ( 0.82,1.35 )  1.14 ( 0.92,1.42) | 98  93  81 | 0.12  0.68  0.23 | 0.12 |
| Sample size  ≥10000  ＜10000 | 1.10 ( 0.98,1.25 )  1.17 ( 1.01,1.35 )  0.95 ( 0.86,1.04 ) | 96  98  0 | 0.12  0.03  0.27 | 0.12 |
| Population  General population  Clinical population | 1.10 ( 0.98,1.25 )  1.21 ( 1.03,1.42 )  0.95 ( 0.87,1.04 ) | 98  99  0 | 0.12  0.02  0.29 | 0.12 |
| Exposure Measurement  Scale  Clinical diagnosis | \| 1.12 ( 0.98,1.28 )  1.09 ( 0.76,1.58 )  1.14 ( 1.95,1.36 ) \| \| --- \| | 98  86  99 | 0.09  0.15  0.64 | 0.09 |

CI: confidence intervals

**Supplementary Fig. 3**. Sensitivity analyses of association between depression and new-onset AF by omitting one study at once
